# Copper-dependent amino oxidase 3 governs selection of metabolic fuels in adipocytes

**DOI:** 10.1371/journal.pbio.2006519

**Published:** 2018-09-10

**Authors:** Haojun Yang, Martina Ralle, Michael J. Wolfgang, Neha Dhawan, Jason L. Burkhead, Susana Rodriguez, Jack H. Kaplan, G. William Wong, Norman Haughey, Svetlana Lutsenko

**Affiliations:** 1 Department of Physiology, Johns Hopkins University, Baltimore, Maryland, United States of America; 2 Department of Genetics, Oregon Health & Science University, Portland, Oregon, United States of America; 3 Center for Metabolism and Obesity Research, Johns Hopkins University, Baltimore, Maryland, United States of America; 4 Department of Biological Chemistry, Johns Hopkins University, Baltimore, Maryland, United States of America; 5 Department of Biological Sciences, University of Alaska Anchorage, Anchorage, Alaska, United States of America; 6 Department of Biochemistry and Molecular Genetics, University of Illinois at Chicago, Chicago, Illinois, United States of America; 7 Department of Neurology, Johns Hopkins University, Baltimore, Maryland, United States of America; Duke University, United States of America

## Abstract

Copper (Cu) has emerged as an important modifier of body lipid metabolism. However, how Cu contributes to the physiology of fat cells remains largely unknown. We found that adipocytes require Cu to establish a balance between main metabolic fuels. Differentiating adipocytes increase their Cu uptake along with the ATP7A-dependent transport of Cu into the secretory pathway to activate a highly up-regulated amino-oxidase copper-containing 3 (AOC3)/semicarbazide-sensitive amine oxidase (SSAO); in vivo, the activity of SSAO depends on the organism’s Cu status. Activated SSAO oppositely regulates uptake of glucose and long-chain fatty acids and remodels the cellular proteome to coordinate changes in fuel availability and related downstream processes, such as glycolysis, de novo lipogenesis, and sphingomyelin/ceramide synthesis. The loss of SSAO-dependent regulation due to Cu deficiency, limited Cu transport to the secretory pathway, or SSAO inactivation shifts metabolism towards lipid-dependent pathways and results in adipocyte hypertrophy and fat accumulation. The results establish a role for Cu homeostasis in adipocyte metabolism and identify SSAO as a regulator of energy utilization processes in adipocytes.

## Introduction

Cu is required for numerous cellular functions, and the loss of Cu homeostasis is incompatible with life [1, 2]. Cu-dependent enzymes critically contribute to mitochondria respiration, cellular defense against oxygen radicals, angiogenesis, wound healing, biosynthesis of neuromodulators, and many other processes [3]. Increasing evidence points to a tight functional link between Cu homeostasis and lipid metabolism. Cu accumulation in the liver alters the tissue levels of triglyceride and cholesterol [4, 5], and, reciprocally, excess fat decreases the hepatic Cu content [6, 7]. Human patients with nonalcoholic fatty-liver disease (NAFLD) and dyslipidemia show Cu deficiency [8, 9], whereas rats fed with a Cu-deficient diet develop insulin resistance and steatosis [10]. Recent studies also suggest that Cu modulates processing of chylomicrons in the intestine [11] and have a signaling role in regulation of lipolysis [12].

Despite this evidence, the role of Cu in adipocyte biology remains largely unknown. Like most cells, adipocytes need Cu for mitochondrial respiration and protection against oxygen radicals. Additionally, adipocyte-specific requirements for Cu are indicated by the presence at the adipocyte plasma membrane of an abundant Cu-dependent enzyme semicarbazide-sensitive amine oxidase (SSAO, also known as amino-oxidase copper-containing 3 [AOC3]/vascular adhesion protein 1 [VAP-1]) [13, 14]. SSAO expression is highest in white fat tissue, and the levels of SSAO mRNA increase significantly during adipocyte maturation [13, 14]. The high expression of SSAO specifically in adipocytes may contribute to characteristic functions of these cells. However, neither the adipocyte-specific function(s) of SSAO nor the role of Cu homeostasis in regulation of SSAO-dependent processes is evident from the currently available data.

Inactivation of SSAO in mice alters leukocyte infiltration of adipose tissue and is associated with mild obesity [15, 16]. Studies in mice also found that administration of SSAO substrate benzylamine improves glucose tolerance and reduces body weight gain [17]. Importantly, adipose SSAO is primarily responsible for these effects [18], but the associated mechanisms remain unclear. Furthermore, none of the studies so far has taken into consideration the fact that significant changes in SSAO levels require adjustments in the availability of Cu, which is essential for structural maturation and enzymatic activity of SSAO [19]. We hypothesized that Cu handling in adipocyte is set to accommodate a SSAO function, which contributes to important adipocyte-specific processes. In testing this hypothesis, we discovered that SSAO controls the network of pathways involved in selection and utilization of metabolic fuels in adipocytes. We also found that this activity of SSAO depends on a cellular/body Cu status and is especially important during adipocyte differentiation into mature fat cells. These results pave the way to further mechanistic studies on the role of Cu and SSAO in normal adipocyte physiology and in metabolic diseases.

## Results

### Cu metabolism is up-regulated during adipocytes differentiation

**Fig 1:**
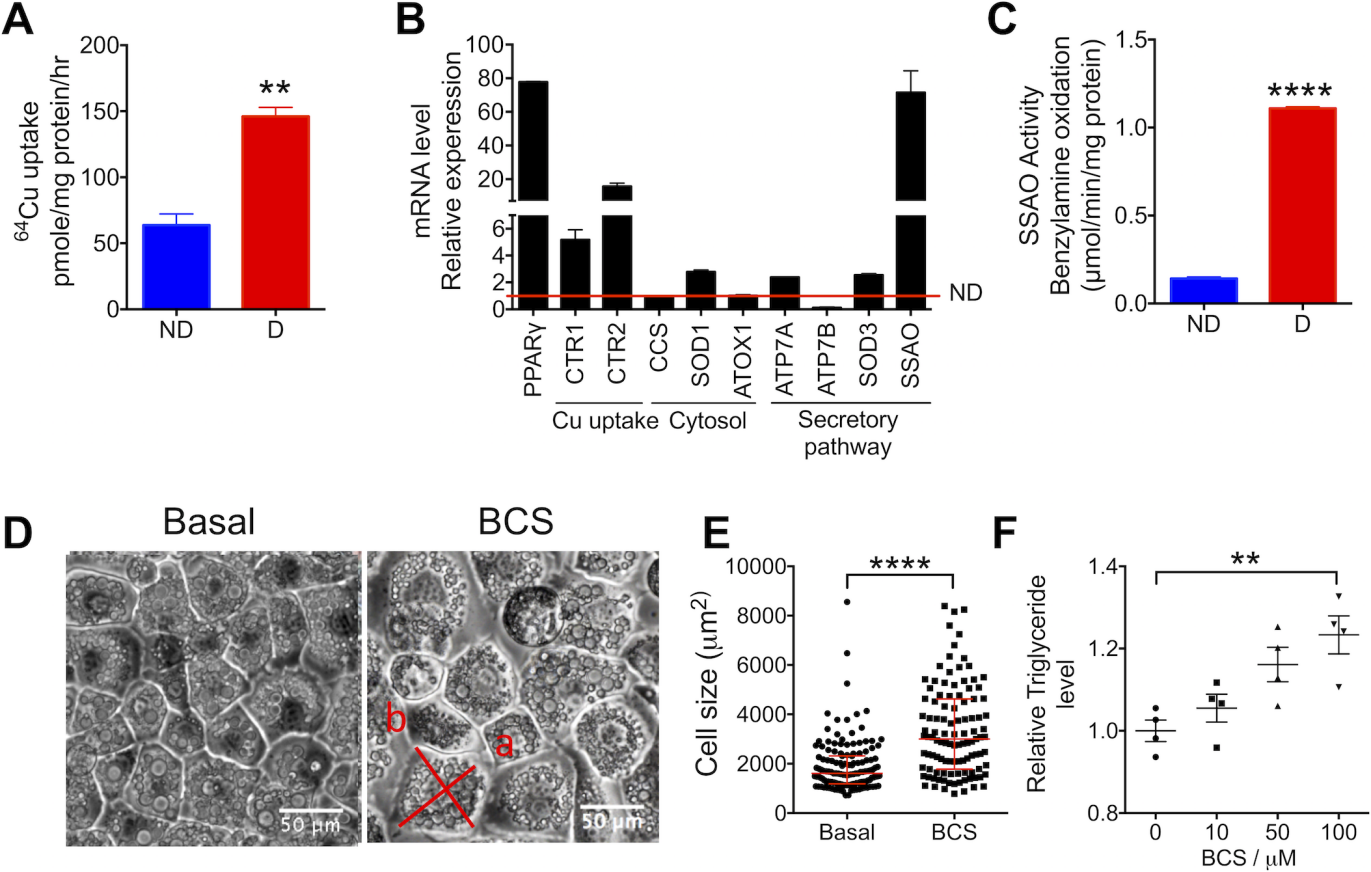
Cu uptake and utilization are increased in differentiated 3T3-L1 adipocytes and contribute to cells morphology and function. (A) Analysis of radioactive ^64^Cu uptake by nondifferentiated (“ND”) and differentiated (“D”) 3T3-L1 cells shows an increased uptake upon adipocyte differentiation (*n* = 3). (B) Changes in the mRNA levels of major Cu-binding proteins as an indirect measure of Cu utilization upon adipocyte differentiation; the red line indicates the mRNA levels in undifferentiated cells (*n* = 3). (C) SSAO activity in differentiated (“D”) cells compared to nondifferentiated (“ND”) 3T3-L1 cells (*n* = 3). (D) Bright-field images of 3T3-L1 cells differentiated in the absence or presence of 50-μM BCS illustrate an increase in cell size upon Cu depletion; “a”—the major cell axe, i.e., “length,” and “b” is the minor cell axe, i.e., “width.” (E) Size distribution of cells differentiated under basal conditions (*n* = 110) and in the presence of 100-μM BCS (*n* = 139). (F) The triglyceride levels in cells treated with BCS (0–100 μM) during differentiation (*n* = 4) compared to basal conditions; triglyceride levels were normalized to protein concentration, and then these values were compared to the triglyceride values at basal conditions; the ratio is plotted. Underlying data can be found in S1 Data; Student’s *t* test, ****p < 0.0001, ***p < 0.001, **p < 0.01, *p < 0.05, ns *p* > 0.05. The data are presented as mean ± SEM and median ± IQR for cell distribution. Atox1, antioxidant 1 copper chaperone; BCS, bathocuproine disulfonate; CTR1, Cu transporter 1; CTR2, Cu transporter 2; CCS, copper chaperone for superoxide dismutase; Cu, copper; ns, not significant; PPARγ, peroxisome proliferator–activated receptor gamma; SOD1, superoxide dismutase 1; SOD3, superoxide dismutase 3; SSAO, semicarbazide-sensitive amine oxidase.

To understand the role of Cu homeostasis in fat-storing cells, we used 3T3-L1 adipocytes. We first examined whether cellular Cu homeostasis changes when 3T3-L1 preadipocytes differentiate into mature adipocytes. Radioactive Cu uptake was 2-fold higher in differentiated adipocytes compared to preadipocytes (146.1 ± 11.8 pmol/mg protein/h versus 63.78 ± 14.6 pmol/mg protein/h; Fig 1A). To understand how this extra Cu is used, we measured the mRNA levels for main Cu-binding proteins before and after adipocyte differentiation; peroxisome proliferator–activated receptor gamma (PPARγ) was used as a differentiation marker and a control. The mRNA levels for the copper uptake transporters 1 and 2 (CTR1 and CTR2) were increased upon differentiation, consistent with a higher Cu uptake. The mRNA for superoxide dismutase 1 (SOD1; the main Cu-binding enzyme in the cytosol) was increased 3-fold in response to differentiation, whereas the transcripts of the cytosolic copper chaperone for superoxide dismutase (CCS) and antioxidant 1 copper chaperone (Atox1) were not significantly changed (Fig 1B). The abundance of mRNAs for plasma membrane Cu-dependent enzymes, superoxide dismutase 3 (SOD3) and SSAO, also increased upon differentiation. The almost 70-fold up-regulation of SSAO mRNA was particularly striking (Fig 1B). The SSAO activity was markedly elevated upon adipocyte differentiation in agreement with the strong up-regulation of mRNA levels (Fig 1C). Thus, adipocyte differentiation is associated with an increased utilization of Cu, especially by SSAO.

SSAO requires Cu for the formation of its characteristic topaquinone cofactor as well as catalytic activity per se (i.e., for the oxidation of primary amines). The Cu-dependent functional maturation of SSAO takes place within the secretory pathway and requires delivery of Cu from the cytosol. In all mammalian cells, this transfer of Cu into the secretory pathway is mediated by the Cu-transporting ATPases ATP7A and/or ATP7B; which of these 2 ATPases operates in adipocytes is not known. The marked increase in SSAO expression/activity upon adipocyte differentiation suggested that one or both Cu-transporting ATPases could be up-regulated in differentiated adipocytes. We found that the ATP7A mRNA levels increased 2.4-fold during adipogenesis, whereas ATP7B mRNA decreased approximately 10-fold, and the protein was not detectable (Fig 1B, S1 Fig). Therefore, ATP7A is the primary Cu transporter working in mature adipocytes and is significantly up-regulated upon adipocyte differentiation.

### Cu deficiency is associated with adipocyte hypertrophy

To determine whether Cu is necessary for adipocyte differentiation and/or function, we used Cu chelators to limit Cu availability during adipogenesis. A membrane-impermeable chelator bathocuproine disulfonate (BCS), which limits Cu entry into cells, was used throughout the differentiation procedure. At the end of differentiation (day 9), the BCS-treated adipocytes were visibly larger (Fig 1D). We measured all adipocytes within a 1-mm^2^ area and found that the median size of Cu-deficient cells was significantly higher compared to the size of control cells that were differentiated in a regular medium. The analysis of size distribution showed a large increase in a number of cells in the upper quartile of values (3,004.3 μm^2^ versus 1,609.1 μm^2^; Fig 1E). We considered that adipocyte hypertrophy is usually associated with an elevated triglyceride content and compared triglyceride levels for control and BCS-treated (Cu-depleted) cells. In BCS-treated adipocytes, the triglyceride levels were significantly higher compared to cells differentiated without BCS addition; increase in triglycerides correlated with an increase in BCS concentrations (Fig 1F). Therefore, Cu limitation is associated with adipocyte hypertrophy and lipid accumulation.

### Decrease in Cu transfer to the secretory pathway induces adipocyte hypertrophy

**Fig 2:**
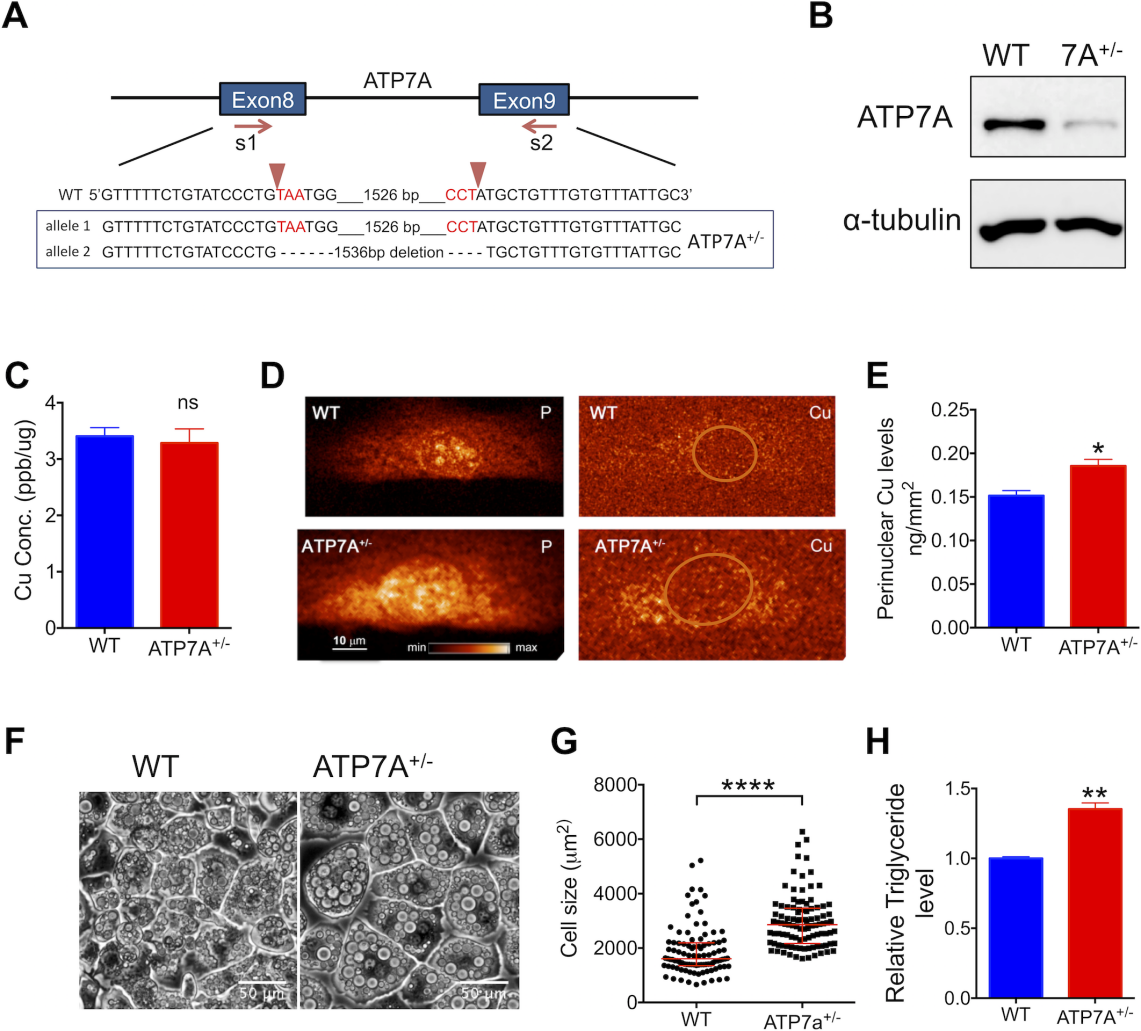
3T3-L1-ATP7A^+/−^ adipocytes are enlarged and have increased triglyceride levels. (A) Schematic of the Cas9/sgRNA targeting of ATP7A. The sgRNA-targeting sequence is shown; the PAM sequence is labeled in red. The results of sequencing of ATP7A genomic regions are shown below the WT sequence. (B) Protein levels of ATP7A in undifferentiated WT and ATP7A^+/−^; α-tubulin is a loading control. (C) The total Cu levels in undifferentiated WT and ATP7A^+/−^ and ATP7A^−/−^ cells measured using atomic absorption spectroscopy (*n* = 3). (D) The XFM images of phosphate (“P”), used as a control, and Cu in undifferentiated WT and ATP7A^+/−^ cells show differences in the intracellular distribution of Cu (*n* = 3); nuclei are indicated by orange circles. (E) The quantitative analysis of fluorescence intensity in the perinuclear region containing bright Cu puncta in WT and ATP7A^+/−^ cells (*n* = 8, 3 respectively). (F) The bright-field images of differentiated WT and ATP7A^+/−^ cells at day 9 illustrate enlargement of cells with down-regulated ATP7A. (G) Distribution of cell sizes and (H) triglyceride levels for WT (*n* = 98; *n* = 3) and ATP7A^+/−^ adipocytes (*n* = 101; *n* = 3); triglyceride levels were normalized to the protein levels, and these values were compared to WT cells’ values taken as 1; the ratio is plotted. Underlying data can be found in S1 Data; Student’s *t* test, ****p < 0.0001, ***p < 0.001, **p < 0.01, *p < 0.05, ns *p* > 0.05. The data are presented as mean ± SEM, and median ± IQR for cell distribution. Cas9, CRISPR-associated 9; Cu, copper; ns, not significant; PAM, protospacer-adjacent motif; sgRNA, single guide RNA; WT, wild type; XFM, X-ray fluorescence microscopy.

Cellular Cu is utilized primarily in the mitochondria, cytosol, and the secretory pathway. The 70-fold increase in SSAO levels during adipogenesis was much higher than the 3-fold increase in the cytosolic SOD1 (Fig 1B) and could exceed the increase in the abundance of mitochondria proteins [20]. Thus, SSAO is a very important “Cu user” in differentiated adipocytes. We hypothesized that the BCS-induced adipocyte hypertrophy and triglyceride accumulation reflect insufficient Cu availability to this enzyme within the secretory pathway. To test this hypothesis, we down-regulated the Cu-transporter ATP7A using clustered regularly interspaced short palindromic repeat (CRISPR)/CRISPR-associated 9 (Cas9)-mediated genome editing. Two single guide RNAs (sgRNAs) were designed to target the exon 8 and exon 9 of ATP7A gene, respectively (Fig 2A). Transfection of cells with these sgRNAs and subsequent selection yielded the 3T3-L1-ATP7A^+/−^ cell line. In these cells, one *Atp7a* allele had a 1,536-bp deletion generated by a simultaneous cutting directed by both sgRNAs; the second allele was not affected. Deletion within one allele of *Atp7a* gene is associated with a lower ATP7A protein levels compared to the wild-type (WT) 3T3-L1-cells (Fig 2B).

To evaluate the functional consequences of ATP7A down-regulation, we characterized the intracellular distribution of Cu, adipocyte size, and triglyceride levels in control and ATP7A^+/−^ cells. In ATP7A^+/−^ preadipocytes, the total Cu content was similar to control 3T3-L1 cells (Fig 2C), but the intracellular distribution of Cu was changed (Fig 2D and 2E). The X-ray fluorescence microscopy (XFM) revealed the puncta-like Cu accumulations in the perinuclear region of ATP7A^+/−^ cells (Fig 2E), presumably due to a diminished transfer of Cu into the secretory pathway. The ATP7A^+/−^ preadipocytes were able to differentiate, but the median size of differentiated ATP7A^+/−^ adipocytes was 80% larger than the size of control adipocytes (Fig 2F)—i.e., 2,856.1 μm^2^ versus 1,608.1 μm^2^ (Fig 2G). Furthermore, the triglyceride levels were elevated in ATP7A^+/−^ adipocytes compared to WT (Fig 2H). The similarity between the phenotype of ATP7A^+/−^ cells and the effects of the BCS-induced Cu deficiency suggested that in both conditions, adipocyte hypertrophy was caused primarily by insufficient Cu levels within the secretory pathway.

### Systemic Cu deficiency inhibits SSAO activity

**Fig 3:**
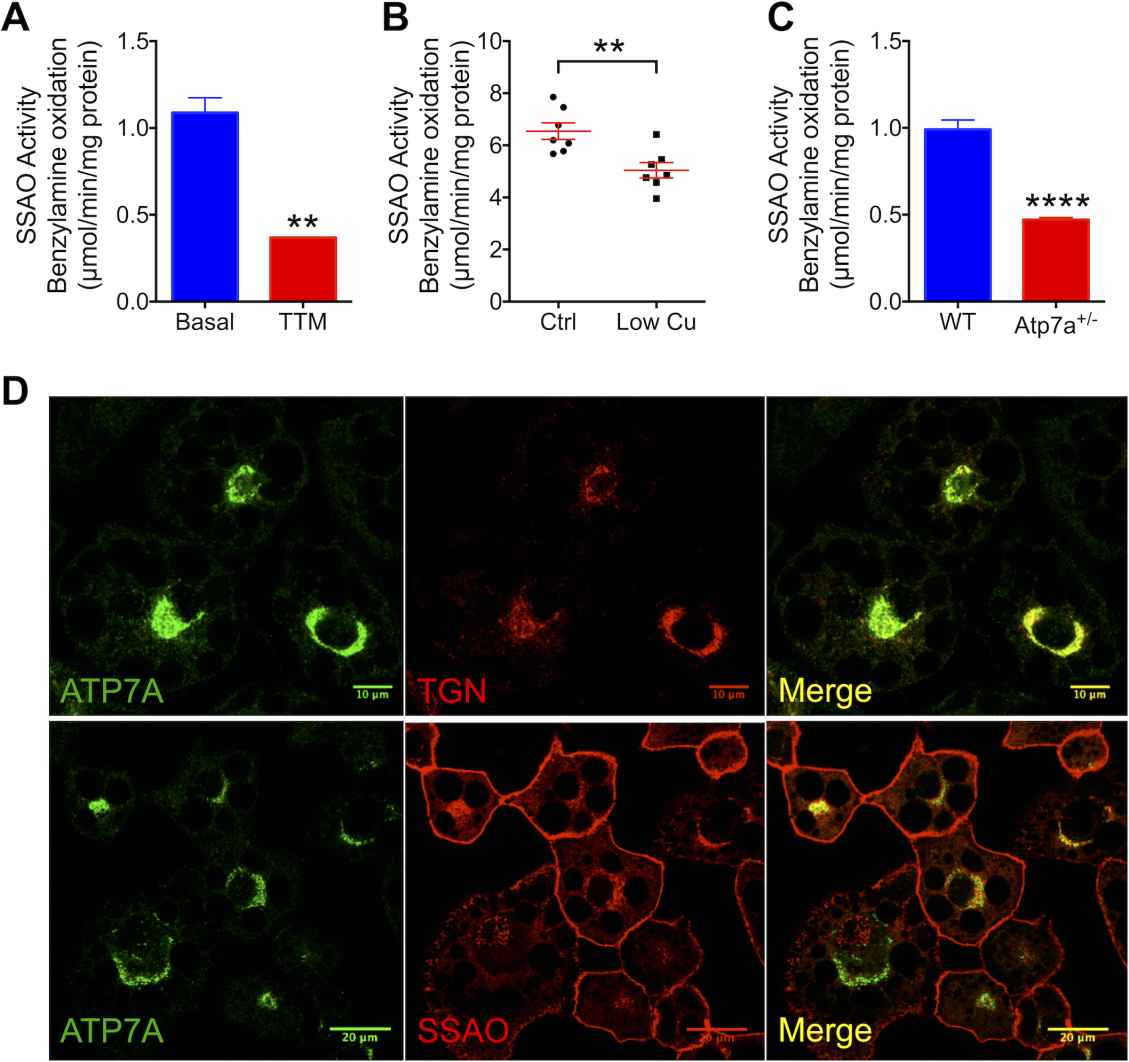
ATP7A-mediated transport of Cu into the secretory pathway is essential for SSAO activity. (A) Cu limitation with 10 μM TTM for 48 h decreases the SSAO activity (*n* = 3). (B) SSAO activity in the epididymal adipose tissue from 13-wk-old male rats fed with Cu-adequate or low-Cu diet for 8 wk (*n* = 7). (C) Down-regulation of ATP7A in ATP7A^+/−^ adipocytes causes a decrease in SSAO activity (*n* = 4). (D) Immunocytochemistry shows that SSAO transits the ATP7A-containing compartment on its way to the plasma membrane. Top: ATP7A (green) is localized to the TGN, as evidenced by its colocalization with the TGN marker Syn6 (red); Bottom: SSAO (red) is present at the plasma membrane and inside the cells, where it colocalizes with ATP7A (green). Underlying data can be found in S1 Data; Student’s *t* test, ****p < 0.0001, ***p < 0.001, **p < 0.01, *p < 0.05, ns *p* > 0.05. Cu, copper; ns, not significant; SSAO, semicarbazide-sensitive amine oxidase; Syn6, syntaxin 6; TGN, *trans*-Golgi network; TTM, tetrathiomolybdate; WT, wild type.

In vitro, the dependence of SSAO activity on Cu cofactor has been firmly established. However, how changes in Cu levels in cells influence SSAO expression and activity is not known. We found that the chelation of intracellular Cu by a membrane-permeable tetrathiomolybdate (TTM) decreases the activity of SSAO (Fig 3A) without affecting SSAO or ATP7A expression (S2 Fig). To determine the impact of Cu homeostasis on SSAO under more physiological conditions, we characterized the effect of dietary Cu deficiency on the activity of SSAO in white adipose tissue. Male rats were fed a low-Cu diet for 3 mo. The Cu levels in the serum and the livers of the Cu-deficient rats were significantly reduced compared to control, as previously described [21], indicative of systemic Cu deficiency. SSAO activity in the epididymal fat of Cu-deficient rats was significantly lower than in control rats (Fig 3B). Therefore, changes in the organismal Cu status have a direct effect on SSAO function.

### SSAO activity depends on ATP7A-mediated transport of Cu into the secretory pathway

To verify that SSAO acquires its Cu within the secretory pathway from ATP7A, we compared the SSAO activity in differentiated 3T3-L1 cells (control) and ATP7A^+/−^ adipocytes. The SSAO-mediated oxidation of benzylamine was significantly lower in ATP7A^+/−^ adipocytes compared to control adipocytes (Fig 3C), even though the expression levels of SSAO were similar (S2 Fig). We also examined the intracellular localization of ATP7A and SSAO. ATP7A was targeted primarily to the *trans*-Golgi network (TGN), as indicated by colocalization of ATP7A with the TGN marker syntaxin 6 (Syn6; Fig 3D). SSAO was found at the plasma membrane and also intracellularly, where it colocalized with ATP7A (Fig 3D). In ATP7A^+/−^ adipocytes, the localization pattern of SSAO was similar to control cells, and the targeting of SSAO to the plasma membrane was not disrupted (S3 Fig). These results are consistent with the role of ATP7A in transferring Cu to SSAO within the lumen of the TGN, when SSAO traffics through the TGN on its way to the plasma membrane.

### The SSAO activity modulates adipocyte size and triglyceride content

**Fig 4:**
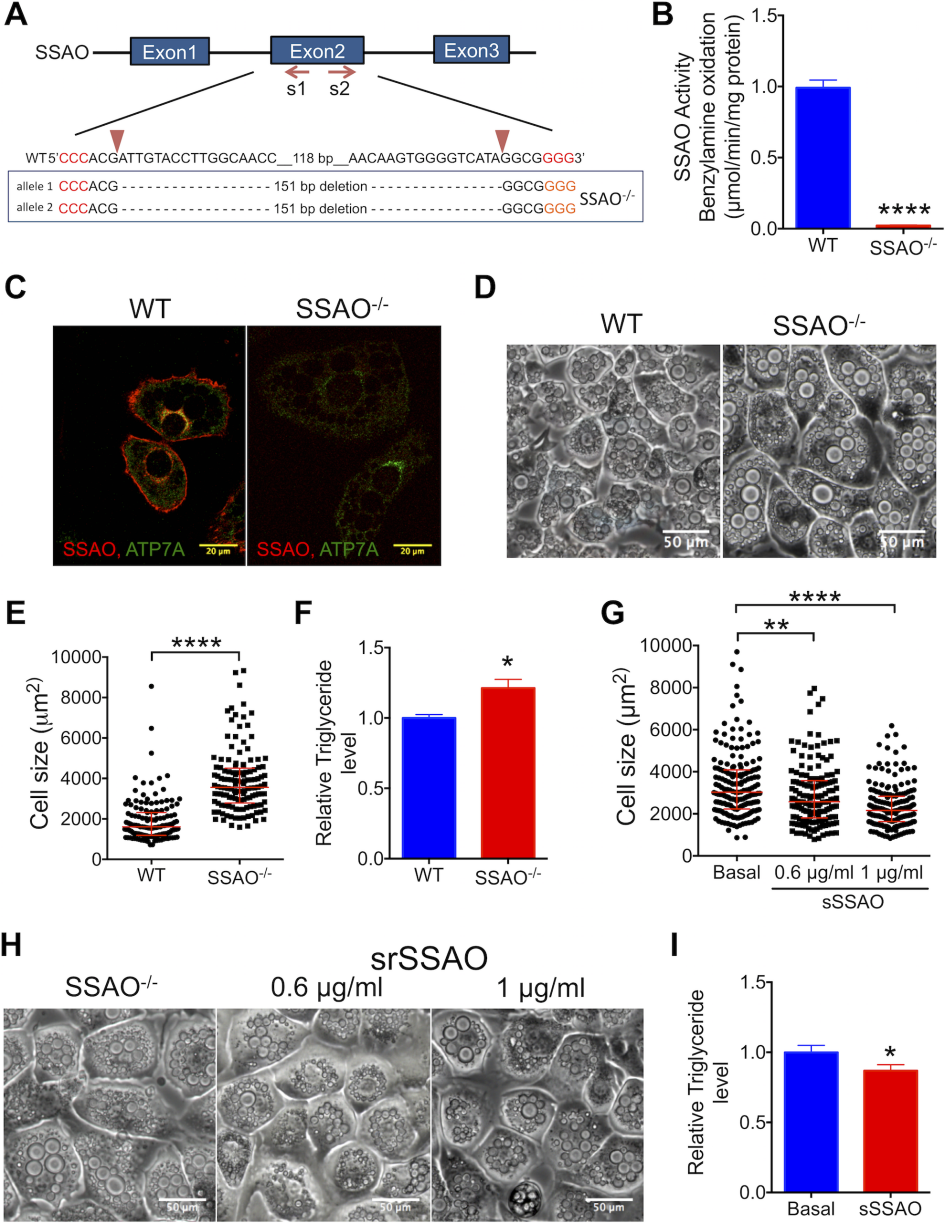
Inactivation of SSAO induces adipocyte hypertrophy. (A) The Cas9/sgRNA-targeting sites in the SSAO gene. The sgRNA-targeting sequence is shown, and the PAM sequence is labeled in red. The results of sequencing of both *AOC3* alleles in SSAO^−/−^ cells are shown under the WT sequence. (B) The SSAO activity in WT and SSAO^−/−^ adipocytes (*n* = 4). (C) Immunostaining of SSAO (red) and ATP7A (green) in the WT and SSAO^−/−^ adipocytes. (D) Bright-field images of differentiated WT and SSAO^−/−^ cells at day 9. (E) The size distribution and (F) triglyceride levels for the WT (*n* = 133; *n* = 3) and SSAO^−/−^ (*n* = 139; *n* = 3) adipocytes (triglyceride levels were normalized to protein levels, and the values for SSAO^−/−^ cells were compared to the WT control; the ratio is plotted). (G) The size distribution and (I) triglyceride levels for SSAO^−/−^ cells in the basal medium (*n* = 178), after treatment with the 0.6 μg/ml sSSAO (*n* = 150; *n* = 3), or 1 μg/ml sSSAO (*n* = 184; *n* = 3); triglyceride levels were normalized to protein levels, and the values for sSSAO-treated cells were compared to values at basal conditions; the ratio is plotted. (H) Effect of sSSAO on the size of SSAO^−/−^ cells at day 9 of differentiation. Underlying data can be found in S1 Data; Student’s *t* test, ****p < 0.0001, ***p < 0.001, **p < 0.01, *p < 0.05, ns *p* > 0.05. The data are presented as mean ± SEM, and median ± IQR for cell distribution. Cas9, CRISPR-associated 9; ns, not significant; PAM, protospacer-adjacent motif; sgRNA, single guide RNA; SSAO, semicarbazide-sensitive amine oxidase; sSSAO, recombinant soluble SSAO; WT, wild type.

Our data suggested that adipocyte hypertrophy and triglyceride accumulation in response to Cu limitation or ATP7A down-regulation could be attributed to the decrease in the Cu-dependent activity of SSAO. To test this hypothesis, we generated a 3T3-L1 SSAO^−/−^ cell line using CRISPR/Cas9 and 2 sgRNAs targeting exon 2 of the *AOC3* gene. The SSAO^−/−^ 3T3-L1 cells had a 151-bp deletion in the exon 2 in both alleles (Fig 4A) and very low SSAO activity (Fig 4B). Immunocytochemistry of SSAO^−/−^ 3T3-L1 adipocytes showed no SSAO staining, in contrast to the WT adipocytes, in which SSAO was easily detected (Fig 4C).

SSAO^−/−^ 3T3-L1 cells were able to differentiate and generate lipid droplets but were highly hypertrophic. The average size of these cells increased more than 2-fold, (Fig 4D): 4,023.3 μm^2^ compared to 1,800 μm^2^ for the WT cells; a similar effect was seen for the distribution of cell sizes (median 3,568.8 μm^2^ SSAO^−/−^ cells compared to 1,609.1 μm^2^ of WT cells; Fig 4E). Lipid droplets were also enlarged (Fig 4D), and the total triglyceride levels were statistically higher in SSAO^−/−^ adipocytes (Fig 4F). To further verify the role of SSAO in adipocyte expansion and lipid elevation, we tested whether the phenotype of SSAO^−/−^ cells could be reversed using a recombinant soluble SSAO (sSSAO) (Pepro Tech, 150–16). Addition of 0.6 μg/ml sSSAO to the cell growth medium during differentiation inhibited enlargement of SSAO^−/−^ cells (Fig 4G and 4H). Treatment with 1 μg/ml sSSAO during differentiation had a stronger effect and also reduced the triglyceride levels in SSAO^−/−^ adipocytes (Fig 4I). The WT cells were not affected by extra sSSAO (S4 Fig). Taken together, these results illustrate that the sSSAO limits adipocyte hypertrophy. We have also found that the hypertrophy of SSAO^−/−^ cells can be rescued by the sSSAO only during adipogenesis. Rescue is not observed after differentiation is completed (S5 Fig). This result indicates that the key SSAO-dependent events, which influence adipocytes properties, occur before cells are fully differentiated.

### Comparative proteomics reveals a regulatory role for SSAO in energy homeostasis

**Fig 5:**
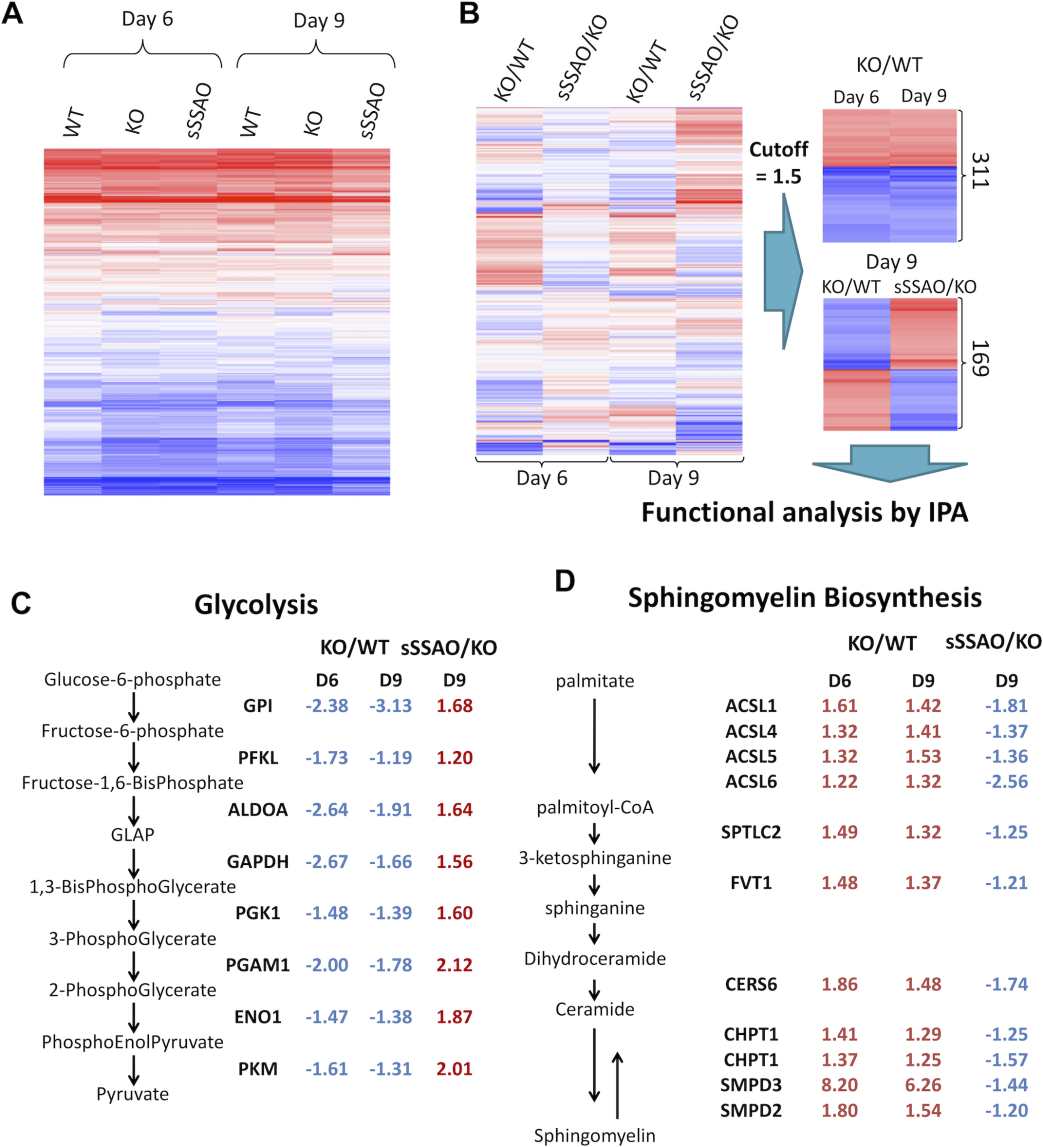
Comparative proteomics reveals a regulatory role for SSAO in energy homeostasis. (A) A heat map of protein abundance in each sample relative to day 0. The log ratios for all 6,098 proteins (protein levels relative to day 0) are color coded, with the blue color indicating negative values and the red color indicating positive values. (B) Left: a heat map comparing abundance of all identified proteins in SSAO^−/−^ cells to those in WT cells (KO/WT) and proteins in SSAO^−/−^ cells without and with treatment with the sSSAO (sSSAO/KO) at day 6 and day 9. At both day 6 and day 9, the changes caused by SSAO inactivation (KO/WT) are reversed by addition of sSSAO (sSSAO/KO); the effect is more complete at day 9. Right: (top panel) the heat map for 311 proteins that show similar changes in their abundance in SSAO^−/−^ cells when compared to WT (KO/WT) at both day 6 and day 9 and (bottom panel) the heat map of 169 proteins deregulated in SSAO^−/−^ cells and reversely changed by treatment with sSSAO (sSSAO/KO); data shown for day 9; 1.5-fold cutoff. (C) The fold change in the levels of proteins within the glycolysis pathway and (D) the sphingomyelin synthesis pathway. The ratios are shown for the SSAO^−/−^ cells versus WT cells (KO/WT) at day 6 (“D6”) and day 9 (“D9”) and for SSAO^−/−^ cells treated with the sSSAO compared to nontreated cells (sSSAO/KO) at day 9. (Red indicates up-regulation, and blue indicates down-regulation.). ACSL1, acyl-coenzyme A synthetase long-chain family member 1; ACSL4, acyl-coenzyme A synthetase long-chain family member 4; ACSL5, acyl-coenzyme A synthetase long-chain family member 5; ACSL6, acyl-coenzyme A synthetase long-chain family member 6; CERS6, ceramide synthase 6; CHPT1, choline phosphotransferase 1; ENO1, enolase 1; FVT1, follicular variant translocation protein 1; GAPDH, glyceraldehyde 3-phosphate dehydrogenase; GLAP, glyceraldehyde 3-phosphate; GPI, glucose-6-phosphate isomerase; KO, knockout; PFKL, phosphofructokinase, liver type; PGAM1, phosphoglycerate mutase 1; PGK1, phosphoglycerate kinase 1; PKM, pyruvate kinase; SMPD2, sphingomyelin phosphodiesterase 2; SMPD3, sphingomyelin phosphodiesterase 3; SPTLC2, serine palmitoyltransferase long chain base subunit 2; SSAO, semicarbazide-sensitive amine oxidase; sSSAO, recombinant soluble SSAO; WT, wild type.

To identify the mechanism driving the SSAO effects on adipocytes, we utilized the isobaric labeling with the tandem mass tags (TMTs) mass spectrometry to assess proteomic changes in the presence and absence of SSAO. We characterized the proteomes of SSAO^−/−^ cells (knockout [KO]), WT cells, and SSAO^−/−^ cells treated with the sSSAO at 3 different time points during adipocyte differentiation. The expression of SSAO is up-regulated after day 4 during in vitro adipogenesis [22]. Therefore, we analyzed cell proteomes prior to adipocyte differentiation (day 0), at day 6 (after SSAO expression is induced), and at day 9, when adipocytes are fully differentiated (Fig 5A). The analysis identified 6,112 distinct proteins, which were detected in all samples with the false discovery rate (FDR) of 0.01 (S2 Data). The protein abundance at day 6 or day 9 was normalized to the protein levels in preadipocytes (day 0), and the protein abundance profiles were generated. Our mass spectrometry data for the WT adipocytes were in good agreement with the previous studies that analyzed changes in protein abundance upon differentiation [20]. As previously reported, we observed a significant increase in mitochondria proteins upon adipocytes’ differentiation (S2 Data) [20]; also as expected, the protein levels of SSAO were increased (S2 Data). Comparison of the profiles for WT and SSAO^−/−^ cells demonstrates that the overall cell differentiation program was not drastically affected by SSAO inactivation, consistent with the ability of SSAO^−/−^ cells to differentiate and form lipid droplets (Fig 5A).

At the same time, distinct changes in response to SSAO loss/additions were apparent (Fig 5B). Altogether, 311 proteins were either up- or down-regulated more than 1.5-fold in SSAO^−/−^ cells at both day 6 and day 9 (Fig 5B, S1 Table). Significantly, these changes were partially or fully reversed by treatment with the sSSAO. The recovery was most pronounced at day 9 (Fig 5B). At this time, the abundance of 169 proteins was changed more than 1.5-fold in SSAO^−/−^ cells and then reversed by treatment with sSSAO (Fig 5B, S1 Table). This set of 169 proteins was defined as an SSAO-dependent proteome and subjected to the pathway analysis using Ingenuity. The analysis identified glycolysis as the pathway that was most significantly changed (down-regulated and then rescued) in response to changes in SSAO activity (Fig 5C). Other down-regulated (and SSAO-rescued) proteins belonged to the pathways involved in pentose phosphate utilization, triacylglycerol degradation, and Hippo and phosphoinositide 3-kinase (PI3K)/Akt signaling (Table 1). In contrast, the abundance of proteins involved in sphingomyelin metabolism, cholesterol biosynthesis, stearate biosynthesis, and ceramide signaling was increased in SSAO^−/−^ cells at both day 6 and day 9 and was down-regulated by additions of sSSAO (Fig 5D). Taken together, the changes in the adipocytes proteome suggested that the enzymatic activity of SSAO during differentiation establishes a balance between the pathways involved in glucose and lipid processing (i.e., energy utilization).

**Table 1:** The SSAO-dependent pathways identified by ingenuity pathway analysis.

Pathways deregulated and rescued at day 9	-log(*p*-value)	Pathways deregulated at day 6 and day 9	-log(*p*-value)
Glycolysis I	5.97	Glycolysis I	4.56
Pentose phosphate pathway	3.12	Sphingomyelin metabolism	3.84
Superpathway of cholesterol biosynthesis	2.98	Pancreatic adenocarcinoma signaling	3.58
Stearate biosynthesis I	2.49	Ceramide signaling	3.47
HIPPO signaling	2.44	PI3K/AKT signaling	3.41
Cholesterol biosynthesis I/II/III	2.42	UDP-N-acetyl-D-galactosamine biosynthesis II	3.38
Methylglyoxal degradation III	2.24	Glycerol-3-phosphate shuttle	2.94
Triacylglycerol degradation	2.18	Ubiquinol-10 biosynthesis	2.79
Glutamine biosynthesis I	2.14	Stearate biosynthesis I	2.59

The pathways deregulated in SSAO^−/−^ cells and rescued by incubation with a recombinant soluble SSAO (left panel). The pathways commonly altered SSAO^−/−^ cells at day 6 and day 9 of differentiation are listed in the right panel. Abbreviations: PI3K, phosphoinositide 3-kinase; SSAO, semicarbazide-sensitive amine oxidase.

### Glucose uptake and de novo fatty acid synthesis are inhibited in SSAO^−/−^ cells

**Fig 6:**
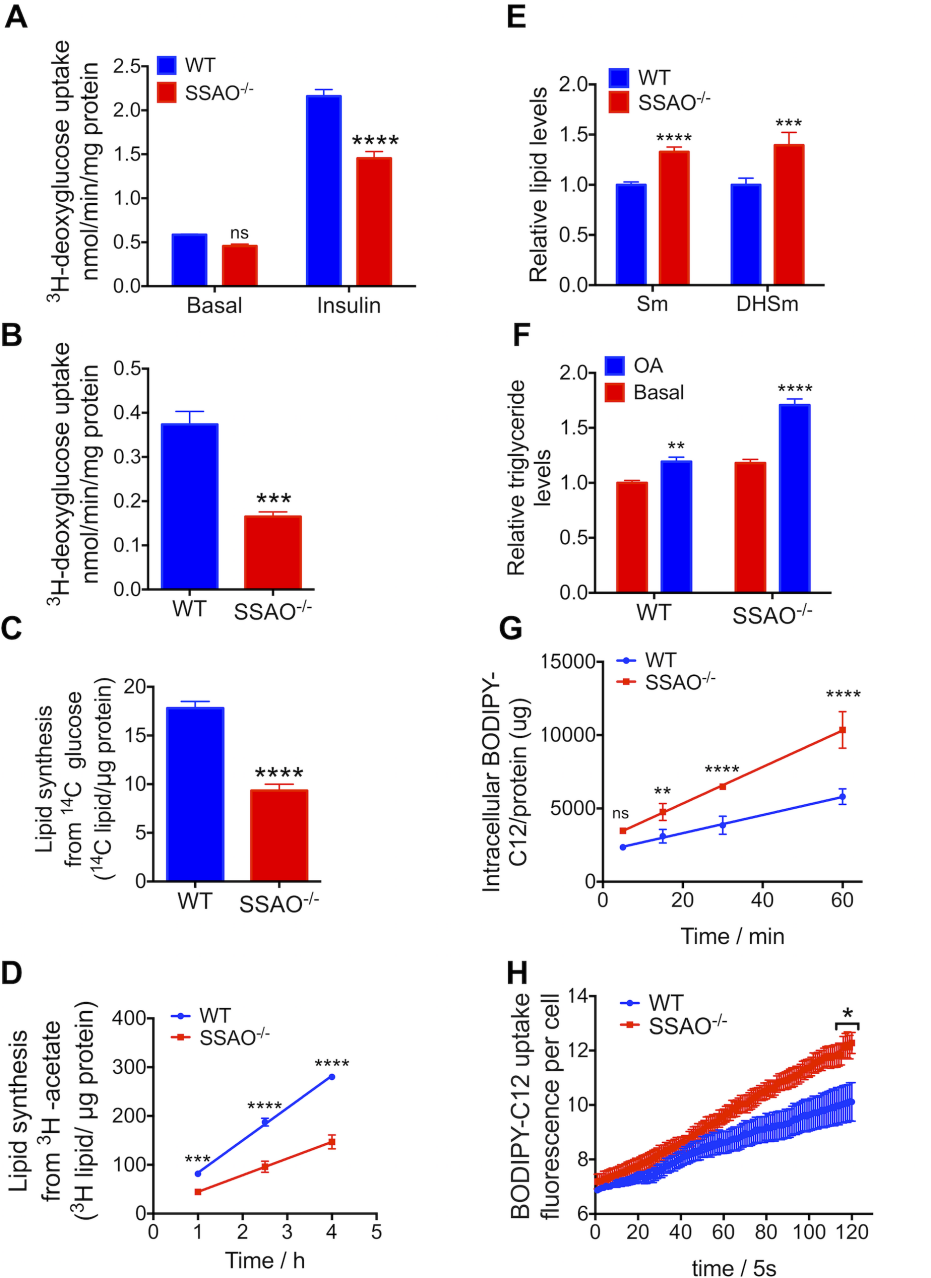
Glucose and fatty acid uptake as well as glucose processing are altered in SSAO^−/−^ cells. (A) The ^3^H-deoxyglucose uptake by WT and SSAO^−/−^ adipocytes with or without 100 nmol/L insulin (*n* = 4) after 2-h fasting in a serum-free low-glucose medium. (B) The ^3^H-deoxyglucose uptake by WT and SSAO^−/−^ adipocytes incubated in a regular growth medium without fasting (*n* = 4). (C) Lipogenesis from ^14^C-glucose in the WT and SSAO^−/−^ adipocytes, overnight incubation (*n* = 6). (D) De novo lipogenesis from ^3^H-acetate in WT and SSAO^−/−^ adipocytes after 1-h, 2.5-h, and 4-h incubation (*n* = 3). (E) The levels of sphingomyelin (“Sm”) and dihydrosphingomyelin (“DHSm”) in differentiated SSAO^−/−^ adipocytes relative to WT cells. (F) The triglyceride levels in the WT and SSAO^−/−^ cells treated without and with 2 mM oleic acid from day 3 to day 9 of differentiation relative to those levels in WT cells without treatment (*n* = 6). (G) BODIPY-C12 uptake in WT and SSAO^−/−^ adipocytes after 5-, 15-, 30-, and 60-min incubation (*n* = 4). Fluorescence intensity in cell lysate is normalized to protein levels. (H) Single-cell BODIPY-C12 uptake in WT (*n* = 4) and SSAO^−/−^ (*n* = 3) adipocytes in 10 min; triglyceride levels were normalized to protein levels, and these values were compared to values at basal conditions; the ratio is plotted. Underlying data can be found in S1 Data; Student’s *t* test for panel (B) and (C), 2-way ANOVA for other panels, ****p < 0.0001, ***p < 0.001, **p < 0.01, *p < 0.05, ns *p* > 0.05. The data are presented as mean ± SEM. ns, not significant; SSAO, semicarbazide-sensitive amine oxidase; WT, wild type.

In SSAO^−/−^ adipocytes, every enzyme involved in glycolysis (from the synthesis of glucose-6-phosphate to the production of pyruvate) was less abundant compared to WT cells but was up-regulated upon SSAO treatment (Fig 5C). It was previously shown that treating cells with the SSAO substrate induces glucose uptake [23]. Therefore, we hypothesized that the adipocytes lacking SSAO down-regulate glycolysis because they have a reduced glucose uptake. To test this hypothesis, we compared glucose uptake for the WT and SSAO^−/−^ cells. After 2-h fasting in a serum-free low-glucose Dulbecco’s Modified Eagle Medium (DMEM), the basal glucose uptake and uptake in response to stimulation with insulin were evaluated. The basal glucose uptake was only slightly decreased in SSAO^−/−^ adipocytes compared to WT adipocytes. The insulin-dependent glucose uptake, measured under maximum insulin stimulation (i.e., with 100 nm insulin), was significantly lower in SSAO^−/−^ adipocytes (Fig 6A). Interestingly, without fasting, in the regular cell growth medium (DMEM with 10% fetal bovine serum [FBS]), the uptake of glucose by SSAO^−/−^ cells was also decreased by almost 2-fold (Fig 6B). These results suggested that during differentiation, SSAO^−/−^ cells were importing less glucose than the WT cells independently whether insulin was present in the growth medium or not.

In adipocytes, glucose uptake and glycolysis are important primarily for fat storage. Most of the acetyl-CoA, which is converted to fatty acids, is derived from glucose through glycolysis. Therefore, diminished glucose uptake/glycolysis would provide less metabolic fuel for lipid synthesis. To test this prediction, we analyzed the glucose-to-lipid conversion using ^14^C-labeled glucose. Under basal growth conditions, the SSAO^−/−^ adipocytes incorporated significantly less glucose-derived ^14^C label into lipids compared to the WT adipocytes (Fig 6C). We also evaluated the de novo fatty acid synthesis using the ^3^H-labeled acetate and found a significantly lower rate of fatty acid synthesis in SSAO^−/−^ cells compared to WT adipocytes (Fig 6D). The protein levels of fatty acid synthase, the key enzyme involved in de novo fatty acid synthesis, were 1.9-fold lower in SSAO^−/−^ cells (proteomics data, S1 Table). Altogether, both glucose uptake and utilization are down-regulated in SSAO^−/−^ adipocytes; these changes are associated with adjustments in the levels of proteins involved in the corresponding pathways.

### SSAO regulates fatty acid uptake and storage

Several proteins associated with the stearate biosynthesis, sphingomyelin metabolism, and long-chain fatty acid processing were up-regulated in SSAO^−/−^ cells (see example in Fig 5E). To test whether these changes affect cellular lipid balance, we measured levels of sphingomyelins and ceramides using TripleToF 5600 mass spectrometry. Changes were observed for several lipids, suggesting increased metabolic activity; for example, the levels of sphingomyelin and dihydrosphingomyelins were higher in the differentiated SSAO^−/−^ cells compared to WT cells (Fig 6E). Given the decreased de novo fatty acid synthesis but increased metabolic activity in the sphingolipid/ceramide pathways, we hypothesized that the fatty acid uptake in SSAO^−/−^ could be up-regulated to accommodate this metabolic activity. To test this hypothesis, we first measured fatty acid accumulation by treating cells with oleic acid (OA) for 6 days during differentiation (from day 3 to day 9). The SSAO^−/−^ cells accumulated significantly more triglycerides (45% over the basal level) than WT cells (20% over the basal level) (Fig 6F). We then directly compared uptake of the fluorescently labeled fatty acid BODIPY-C12 by WT and SSAO^−/−^ cells. The fatty acid uptake over a 1-h time interval was higher in SSAO^−/−^ cells compared to WT cells (Fig 6G). The initial rate of fatty acid uptake (first 10 min) was also higher (Fig 6H). Lastly, we examined whether these functional changes are associated with changes in the cell proteome. Proteins involved in fatty acid uptake and intracellular compartmentalization—such as fatty acid translocase (CD36), long-chain fatty acid transport protein (FATP), long and very long fatty acid transporter ATP-binding cassette subfamily D member 1 (ABCD1), gamma-butyrobetaine dioxygenase (BBOX1), acyl-coenzyme A synthase (ACSL), and caveolin-1—were elevated in SSAO^−/−^ cells at day 6 and day 9 (S2 Table). Thus, fatty acid uptake, transfer to cell compartments, and metabolism are enhanced in SSAO^−/−^ cells.

## Discussion

This study establishes Cu homeostasis as an important component of adipocyte metabolism. Specifically, we show that Cu transfer to the secretory pathway and activation of SSAO is a prerequisite for establishing a balanced energy utilization in differentiating adipocytes. The relationship between Cu levels and lipid metabolism (especially Cu deficiency) has been repeatedly observed in humans, laboratory animals, poultry, finishing pigs, and cattle [24–26], but the molecular mechanisms remained unclear. Our study demonstrates that Cu deficiency, in vitro and in vivo, directly translates to inhibition of SSAO activity, which triggers specific metabolic changes culminating in adipocyte hypertrophy and increased fat deposition. Significantly, SSAO inhibition is associated with long-lasting changes in specific pathways (glycolysis, de novo lipogenesis, and fatty acid transport/utilization) that cannot be easily reversed post differentiation. It remains to be determined whether the Cu-dependent SSAO activity causes epigenetic changes or modulates cell proteostasis. Nevertheless, it is clear that dramatic up-regulation of SSAO during adipocyte differentiation serves a very important purpose.

SSAO is a highly abundant membrane protein, which contributes up to 2.3% of the total plasma membrane proteins in differentiated adipocytes [27]. This high abundance along with the increased levels of soluble SSAO in obesity attracted early attention; and SSAO has been extensively characterized biochemically and in animals. Our identification of the SSAO-dependent pathways in adipocytes adds mechanistic underpinnings to several previous observations. The insulin-like effect of SSAO substrates [28, 29] is in good agreement with our finding that SSAO activity stimulates glucose uptake/glycolysis by changing abundance of proteins involved in these processes. Similarly, the reported enlargement of white adipose tissue in SSAO^−/−^ mice [16, 30] is consistent with our finding that SSAO^−/−^ adipocytes are bigger and have increased fat content, especially following OA treatment. Up-regulation of fatty acid uptake, increased abundance of lipid trafficking proteins, and changes in signaling lipids, observed in our study, are likely to be responsible for the fat accumulation phenotype.

We have also found that the soluble SSAO is sufficient to reverse cell hypertrophy. This result suggests that the SSAO catalytic domain (activity) rather than the mere presence of the membrane protein is important for cell size. The SSAO-dependent change in the abundance of intermediate filament protein vimentin is likely a result of inhibited insulin signaling [31] and, together with elevated collagen proteins—such as type I, III, IV, V, and VI collagens—may contribute to changes in the SSAO^−/−^ cell size. Other potentially relevant SSAO-dependent changes include an altered abundance of anoctamin 10 (Ano10; a chloride channel that controls cell volume [32]) and thrombospondin 1 (THBS1; an adhesive glycoprotein that mediates cell-to-cell and cell-to-matrix interactions [33]).

Adipose tissue buffers circulating free fatty acids through a fine-tuned cycle of uptake, esterification, and release. In the postprandial period, adipose tissue adsorbs fatty acids liberated by lipoprotein lipase from carriers of dietary fat, such as chylomicrons and very low-density lipoprotein (VLDL). Uptake of free fatty acid has also been reported [34], although it is barely detectable in the fasting/postabsorptive state. SSAO-dependent changes in the abundance of proteins involved in the transport and processing of long-chain fatty acids suggest a potential role for SSAO in regulation of this free fatty acid uptake.

Our study does not provide an obvious answer to how SSAO regulates leukocyte recruitment and inflammatory response (a well-established function of SSAO), because the abundance of proteins directly involved in leukocytes extravasation is not affected by SSAO inactivation. However, inactivation of SSAO causes marked down-regulation of semaphorin 3G (a signaling molecule with a cell-repulsive function) and up-regulation of CD74 (a receptor for macrophage migration inhibitory factor, which promotes immune cell recruitment to the sites of inflammation) [35, 36]. These changes could be relevant to SSAO function in leukocyte recruitment. The SSAO-dependent changes in the oligosaccharyltransferase complex (OSTC; which catalyzes the transfer of high-mannose sugars) may significantly alter properties of cell surface receptors involved in cell–cell recognition/binding. Together, our data suggest that SSAO may act as a molecular link between the metabolic state of adipocytes and their signaling activity and thus be a key player in the obesity-induced inflammation.

Finally, the previously unrecognized function of mammalian SSAO in energy metabolism could be an evolutionally conserved function of this enzyme. In *Escherichia coli*, a Cu-dependent amine oxidase (ECAO) encoded by the tynA gene is a structural homologue of the mammalian SSAO and has similar enzymatic activity. A recent study revealed the role for ECAO in cell energy metabolism [37]. Deletion of tynA/ECAO causes significant metabolic changes, especially in the pathways involved in the conversion of phenylethylamine to acetyl-CoA and oxidation of propionate to pyruvate [37]. High conservation of the Cu transport machinery (the *E*. *coli* Cu transporter CopA is homologous to the mammalian ATP7A) further highlights an early evolutional coupling between Cu homeostasis and the processing of metabolic fuels.

In summary, we have established the role for Cu homeostasis in adipocyte morphology and function and identified SSAO as a regulator of adipocyte mass and metabolism. We show that adipocytes require proper Cu balance and compartmentalization to maintain their size and energy balance. In differentiated adipocytes, Cu transfer into the secretory pathway is regulated by the Cu transporter ATP7A; ATP7A supplies Cu to SSAO, which is highly up-regulated in differentiated adipocytes. Our data suggest a novel mechanistic role of SSAO in selection of metabolic fuels and fat storage. Understanding the role of Cu and SSAO in obesity will help to develop new tools and treatment strategies to reduce obesity and its complications, such as diabetes and fatty liver.

## Methods

### Ethics statement

Animal experiments and husbandry were approved by the University of Alaska Anchorage (UAA) Institutional Animal Care and Use Committee (USDA Assurance #A3710-0) and performed in accordance with United States Public Health Service Policy as documented by *The Guide for the Care and Use of Laboratory Animals 8th Edition*. Eight-wk-old male Wistar rats that had been allowed ad libitum access to Mazuri Rodent Diet (PMI Nutrition, St. Louis, MO, USA) were used in the study. Rats were randomly assigned to one of 2 groups and fed for 5 wk with diets based on the Purified AIN76A formulation, modified with Cu content as described by Tallino and coworkers [21]: Cu deficient (<0.3 mg Cu/kg), Cu adequate (125 mg/kg). Rats were killed by carbon dioxide asphyxiation (consistent with 2013 AVMA guidelines) and exsanguinated, and adipose tissue specimens were frozen in liquid nitrogen and then stored at −80°C until use.

### Cell lines and culture conditions

The 3T3-L1 cells were cultured in high-glucose DMEM (Gibco) supplemented with 10% FBS (Sigma Aldrich) and 1% Penstrep (Gibco) (basal medium). Differentiation protocol for 3T3-L1 cells included growing cells with the basal medium for 2 d after cells reached confluence (d-2); then, cell differentiation was induced by changing the medium to the basal medium with 0.5 mM 3-Isobutyl-1-methylxanthine (IBMX), 1 μM dexamethasone, 10 μg/ml insulin, and 2 μM rosiglitazone (differentiation medium I [DMI]) for 3 d (d0–d3), followed by the basal medium with 10 μg/ml insulin (differentiation medium II [DMII]) for 2 d (d3–d5). On day 5, the DMII medium was changed to basal medium for 3–4 days. Cells at day 2 were defined as undifferentiated adipocytes and cells at day 8 or day 9 as differentiated mature adipocytes (Fig 2A). For various treatments, either 0–100 mM BCS (Sigma) or a human sSSAO (0.6 μg/ml or 1 μg/ml) (Pepro Tech, 150–16) was included into the basal and differentiation media throughout the entire procedure (i.e., from d0 to d8, fresh SSAO was added every 2 d). To limit intracellular Cu, differentiated adipocytes were treated with 10 μM TTM in basal medium for 48 h.

### Cu uptake

The experiment was performed as previously described in [38]: undifferentiated 3T3-L1 cells (d2) or differentiated 3T3-L1 cells (d8–9) were plated in 12-well collagen-coated plates. Prior to measurements, cells were washed twice with PBS. Then, fresh DMEM supplemented with 10% FBS was added to each well, and cells were equilibrated for 30 min at 37°C. Cu uptake was initiated by adding 5 μM CuCl_2_ labeled with trace amounts of ^64^Cu (MIR radiological sciences, Washington University Medical School). Cells were incubated for 30 min at 37°C, and Cu uptake was terminated by addition of ice-cold stop buffer (150 mM NaCl, 5 mM KCl, 2.5 mM MgCl_2_, 25 mM HEPES, pH 7.4, and 10 mM Na_2_EDTA), after which cells were washed 3 times with the ice-cold stop buffer. Cells were lysed with 1 ml of 1N NaOH, and 700 μl of lysates was taken for scintillation counting (Beckman-Coulter LS6500) with Eco-Lume scintillation liquid (ICN Biomedicals #882470). The remaining portion of cell lysates was left until radioactivity decayed, for the determination of total protein concentration in each sample, Bio-Rad Protein Assay (BioRad #500–0006). The ^64^Cu transport measurements were carried out in triplicates. The ^64^Cu content of the initial tracer-containing buffer was determined for the calculation of specific activity, and, following determination of the protein content, Cu uptake was then expressed as picomoles of Cu taken up by the cells per milligram of total cellular protein per minute.

### Immunoblot analysis

Cells were cultured in a 24-well plate, 6-well plate, or 100-mm dish and washed with PBS twice prior to lysis. Cells were lysed with RiPA buffer (Millipore) (with recommended EDTA-free protease inhibitor cocktail [Sigma]) on ice for 1 h, and unsolubilized material was removed by centrifugation at 3,000 g for 15 min. Protein concentration in supernatant was determined by the BCA assay, and 20 μg protein was used for further analysis. Before electrophoretic separation of proteins, each sample was combined with an equal volume of 2 x Laemmli sample containing 5% β-mercaptoethanol. Proteins were then resolved on 8% Laemmli SDS-PAGE and transferred to PVDF membrane at 90 V for 90 min. Primary antibodies used in immunoblotting were rabbit monoclonal anti-ATP7A CT77 (Hycult biotech), mouse monoclonal anti-tubulin (Sigma, T8203), mouse Anti-Na+/K+ ATPase α-1 Antibody (Millipore 06–520), and rabbit Anti-ATP7b antibody [EPR6794] (ab124973). Secondary antibodies were goat polyclonal anti-mouse IgG HRP-conjugate (Santa Cruz, SC-2005) and goat polyclonal anti-rabbit IgG HRP-conjugate (Santa Cruz, SC-2004). All antibodies were used at a dilution of 1:1,000.

### Immunostaining of cultured cells

Cells were cultured on cover slips in a 12-well plate, fixed with ice-cold 1:1 acetone: Methanol solution for 30 s followed by 5-s washing with PBS, and blocked with 1% (w/v) BSA/1% (w/v) gelatin in PBS overnight in 4°C. Primary antibodies used in immunoblotting were mouse monoclonal anti-rabbit anti-ATP7A CT77 (Hycult biotech), mouse monoclonal anti-tubulin (Sigma, T8203), mouse monoclonal anti-SSAO 7–106 (AK 982/02), mouse anti-Syn6 (BD Transduction Laboratories, 610636), and goat polyclonal anti-mouse IgG Alexa488-conjugate (Molecular Probes, A-11001). Secondary antibodies were goat polyclonal anti-rabbit IgG Alexa488-conjugate (Thermo, A11034) and goat polyclonal anti-mouse IgG Alexa488-conjugate (Thermo, A31570). All antibodies were used at a dilution of 1:100. Cells were imaged with a Zeiss LSM 710. Images were processed with ZEN and Image J.

### Cu measurements by atomic absorption

Cells cultured in a 6-well plate were washed, collected, suspended in PBS, and divided into 2 aliquots. One aliquot was pelleted, supernatant removed completely, and then 70% nitric acid was added to the pellet and incubated at 55°C for 30 min. The samples were diluted 40 times with sterile water (VWR) and centrifuged at maximum speed (14,000 g) for 1 min. Cu concentration in the supernatant was measured using atomic absorption spectrometer (AA-6650G, Shimadzu, Columbia, MO, USA). The second aliquot was pelleted, and cells were lysed using RiPA buffer (with EDTA-free protease inhibitor cocktail) on ice for 1 h and centrifuged at 3,000 g for 15 min. Protein concentration of the resulting cleared lysate was determined by BCA assay. A Cu/protein ratio was averaged for each sample in 3 independent samples.

### Quantitative real-time PCR (RT-PCR)

RT-PCR was performed as previously described in [39]; specifically, total RNA was isolated from cells with RNeasy kit (Qiagen), and corresponding cDNA pools were synthesized using Fast-Strand cDNA synthesis kit (Roche). RT–PCR was performed with SYBR green (Applied Biosystems) on an ABI 7500 Sequence Detection System (Applied Biosystems). For ΔΔCt analysis, GAPDH levels were used for normalization. Primers used in this study are listed in the S3 Table.

### CRISPR/Cas9 genomic edited cell line

CRISPR/Cas9 genomic editing and vector (pEF6/V5-His TOPO TA)-expressing Cas9 were previously described in [40, 41]. The sgRNA sequences were designed to target the transmembrane region of ATP7A gene and determined by the CRISPR Design Tool (http://crispr.mit.edu/). Two synthesized sgRNA oligos (Integrated DNA Technology) for each gene (ATP7A target 1: 5′-GTTTTTCTGTATCCCTGTAATGG-3′ and ATP7A target 2: 5′-CCTATGCTGTTTGTGTTTATTGC-3′) were ligated into the pEF6/V5-His TOPO TA vector using In-Fusion HD Cloning Kit (Clontech Laboratories) to produce the Cas9-sgRNA plasmid. The 3T3-L1 cells were transfected with 2 μg Cas9-sgRNA plasmid using Lipofectamine LTX with Plus Reagent (Life Technologies). Transfected cells were selected by growing in the basal medium supplemented with 3 μg/ml blasticidin for 2 wk and were then diluted into a 96-well plate for cell cloning. The individual clones were taken from wells into a larger plate and propagated until the cell number was higher than 1 × 10^6^, at which point cryo-stocks were prepared. Cell clones with down-regulated ATP7A were identified by western blotting of whole-cell lysate. Deletion in ATP7A gene was further confirmed by Sanger sequencing. To do that, genomic DNA was isolated from cells with down-regulated ATP7A using GenElute Mammalian Genomic DNA miniprep Kit (Sigma-Aldrich). PCR was performed using specific primers for the targeted region in ATP7A gene (forward primer: 5′-TCTTAGCCTGAGTGAGATGGTT-3′, reverse primer: 5′-TCCACTATCTTAACAAATGTCACCC-3′). The PCR products were cloned using the TOPO TA cloning Kit (Life Technologies) and transformed into *E*. *coli*. Plasmids containing PCR products were isolated using QIAprep Spin Miniprep Kit (Qiagen) and analyzed at the synthesis and sequencing facility at Johns Hopkins School of Medicine.

The 3T3-L1 adipocytes with inactivated SSAO were generated using similar protocol. Two sgRNA sequences were designed to target exon 2 of SSAO gene using the CRISPR Design Tool (crispr.mit.edu/). (SSAO target 1: 5′-CCCACGATTGTACCTTGGCAACC-3′; SSAO target 2: 5′-AACAAGTGGGGTCATA-GGCGGGG-3′). The cryo-stocks and genomic DNA of samples were prepared. And the SSAO^−/−^ cells were identified by PCR. PCR was performed using primers designed to amplify the sgRNA-targeted region in the SSAO gene (forward primer: 5′- CTGCAGTCAGGTAGATGGCA-3′, reverse primer: 5′- GTGGTGAGTGATCAGA-GGGC-3′). PCR products were examined on an ethidium bromide–stained 10% acrylamide Criterion TBE gel (BioRad). The PCR product from control cells should be 378 bp, and product from cells with the SSAO gene simultaneously cut by 2 sgRNAs should be around 230 bp. Then, the positive PCR products were cloned using the TOPO TA cloning Kit (Life Technologies) and transformed into *E*. *coli*. Plasmids containing PCR products were isolated using QIAprep Spin Miniprep Kit (Qiagen) and sequenced at the synthesis and sequencing facility at Johns Hopkins School of Medicine.

### Glucose uptake

Glucose uptake in 3T3-L1 adipocytes was performed as described [42]. In brief, differentiated adipocytes cultured in a 24-well plate were serum starved in the low-glucose DMEM for 2 h. The medium was then replaced with the Krebs-Ringer-HEPES buffer, and adipocytes were incubated with or without 100 nM insulin for 20 min. Radioactive 2-deoxy-D-[2,6-3H]-glucose (MT1611) (1 **μ**Ci/well) was added, and cells were incubated for 10 min. Uptake was stopped by aspirating the medium, and cells were washed 3 times with the ice-cold PBS buffer. The cells were then lysed with the RiPA buffer, and aliquots were taken for protein content analysis using BCA assay (Pierce). Radioactivity of cell lysates was counted in Ecoscint scintillation mixture (National Diagnostics) using a Beckman LS-6000 liquid scintillation counter, and counts were normalized to protein content. The basal glucose uptake in the standard growth medium was evaluated using the same protocol, except cells were inculcated with 1 **μ**Ci/ml of 2-deoxy-D-[2,6-3H]-glucose for 10 min and 20 min without prior starvation.

### De novo fatty acid synthesis

Differentiated WT and SSAO^−/−^ 3T3-L1 cells cultured in a 24-well plate were labeled with the trace levels (1.0 **μ**Ci) of [^3^H]acetate (Perkin Elmer) for 1 h, 2.5 h, and 4 h. Lipids were extracted with chloroform/methanol using the Folch method [43], and radioactivity was counted by liquid scintillation. Equal amounts of cells seeded in parallel in a 24-well plate were lysed with 1x RiPA buffer for protein content analysis using BCA assay kit (Pierce). The protein amounts of each well between WT and SSAO^−/−^ adipocytes were 151.2 **±** 7.8 **μ**g and 117.1 **±** 2.1 **μ**g (mean **±** SD); the SD among wells is much smaller than the difference between WT and SSAO^−/−^ adipocytes. Radioactivity of cell lysates was counted in Ecoscint scintillation mixture (National Diagnostics) using a Beckman LS-6000 liquid scintillation counter. Radioactivity in each sample was normalized to protein content.

### Lipogenesis

Differentiated WT and SSAO^−/−^ adipocytes cultured in a 24-well plate were labeled overnight with the trace levels (0.3 **μ**Ci/well) of [^14^C] glucose (Perkin Elmer) in DMEM low-glucose medium (1 g/L, ThermoFisher) with 10% FBS (Sigma Aldrich). Lipids were extracted with chloroform/methanol using the Folch method [43], and radioactivity was counted by liquid scintillation. Cell seeded in a 24-well plate at the same time were lysed with 1x RiPA buffer for protein content analysis using BCA assay kit (Pierce). Radioactivity of cell lysates was counted in Ecoscint scintillation mixture (National Diagnostics) using a Beckman LS-6000 liquid scintillation counter. Radioactivity in each sample was normalized to protein content.

### Lipid uptake

Cells were cultured in 96-well plates and differentiated into mature adipocytes as described above. The fluorescent fatty acid analogue 10 μM BODIPY FL C12 was added to the basal medium, and cells were incubated for different periods of time. Uptake was stopped by removing medium and washing cells with cold PBS. Then BODIPY fluorescence (excitation: 500 nm, emission: 510 nm) was measured in a BMG Labtech FLUOstar Omega plate reader. Cells grown on the 35-mm glass-bottom dishes were used to measure fatty acid uptake in live cells. Images were acquired using a Zeiss LSM510 Meta with 488-nm laser and 40× objective. First, the baseline fluorescence was recorded; then, the 4 μM BODIPY FL C12 was added to cells. The fluorescence of BODIPY FL C_12_ was excited at 491 nm, and 530-nm emission was collected. Time series images were taken every 5 s over a period of 10 min. Image J was used to analyze the data.

### XFM

XFM was performed as previously described in [40]: cells were grown on 4 × 4-mm silicon nitride (SiN) membranes (Silson, Northampton, England). To enable cell growth, the SiN membranes were sterilized with UV radiation and incubated with 10 μL sterile 0.01% POLY-L-Lysine solution (Sigma-Aldrich, St Louis, MO, USA) at 37°C. After 30 min, POLY-L-Lysine was removed, and 10 μl of basal media with suspended cells was added to the membrane. Cells were allowed to adhere to the membrane O/N at 37°C, and then basal medium was added to cover the bottom of plate. After 1-d recovery, the membranes were rinsed with PBS, fixed with 4% paraformaldehyde for 30 min at 37°C, rinsed with PBS, and then rinsed with 100 mM isotonic ammonium acetate and water and were air-dried. XFM images were collected on beamline 2-ID-E at the Advanced Photon Source, Argonne National Laboratory, Argonne, IL, USA. SiN membranes were mounted onto kinematic sample holders, and target cells were selected using a light microscope (Leica, Buffolo Grove, IL, USA) equipped with a high precision, motorized x, y-stage (Ludl Electronic Products, Hawthorne, NY, USA). Coordinates of target cells were recorded before mounting the sample on the microprobe stage at the beamline. The microscope coordinates were translated into microprobe coordinates and the cell raster scanned in the x-y plane. The incident X-ray energy was tuned to 10 keV using a Si-monochromator, and the monochromatic beam was focused to 750 × 750 nm using a Fresnel zone plate. The sample was placed at 19° to the incident X-ray beam, and the resulting X-ray fluorescence was collected at 90° using an energy-dispersive 4-element detector (Vortex ME-4, SII Nanotechnology, Northridge, CA, USA). Elemental maps were created by extracting, background subtracting, and fitting the fluorescence counts for each element at each point using the program MAPS [44]. The fluorescent photon counts were translated into μg/cm2 using calibrated X-ray standards (AXO products, Dresden, Germany).

### SSAO activity

Tissue and 3T3-L1 cells were washed with PBS and then homogenized using RiPA buffer (with an EDTA-free protease inhibitor cocktail) on ice for 1 h, and nonsolubilized material was removed by centrifugation at 3,000 g for 15 min. Tissue homogenates were prepared on ice by homogenizing pieces of frozen rat fat tissue in 1 ml of homogenizing buffer (50 mM HEPEs, 1/1,000 Igepal, 150 mM NaCl, 0.25 M sucrose, 0.5 μM AEBSF, and 1:100 protease inhibitor solution). The samples received 30 strokes with each loose and tight-fitting piston in a Dounce homogenizer and were then centrifuged at 700 g for 15 min to remove the debris. The supernatant was collected (whole-cell lysate), and protein concentration in the lysate was determined by BCA assay.

SSAO activity was determined at 37°C by measuring production of hydrogen peroxide using Amplex Red Monoamine Oxidase Assay kit following the manufacturer’s (Molecular Probes, the Netherlands) protocol. Briefly, 25 μg cell lysates or 10 μg tissue homogenates were preincubated for 30 min at 37°C with 100 μM pargyline hydrochloride to inhibit monooxygenase B (MAO-B) activity. The SSAO reaction was then started by the addition of 100 μl reaction buffer (400 μM Amplex Red, 2 U/mL horseradish peroxidase, 2 mM benzylamine, 50 mM sodium phosphate, pH 7.4), and a change in fluorescence (excitation: 530 nm, emission: 590 nm) was monitored every 2 min for 60 min at 37°C in a microplate reader. To determine the background, the identical samples were incubated in parallel with the 1 mM of SSAO inhibitor semicarbazide. Resorufin was used to generate a standard curve for activity calibration. SSAO activity was expressed as pmol/ mg protein/min.

### Triglyceride levels

Cells in a 24-well plate were washed 2 times with PBS and lysed with 80 μl RiPA buffer on ice for 30 min. Cell lysates were vortexed for 30 s, and triglycerides were quantified calorimetrically by measuring glycerol (Infinity Triglycerides, Fisher Diagnostics). Triglyceride levels were expressed as total triglyceride per well. To compare triglyceride in different cell lines, triglyceride levels were normalized to protein concentration. To measure protein concentration, cell lysates were centrifuged at 3,000 g for 15 min at 4°C, and supernatants were used in BCA assay.

### Adipocytes size

Live differentiated 3T3-L1 cells were imaged with a light phase–contrast microscope (Olympus IX51). Image J was used to quantitatively analyze the size of adipocytes. The length of major axe (a) and minor axe (b) of each adipocyte was measured (Fig 3A), and the elliptic area was calculated (1/4π a × b). Cell size distribution was analyzed using all cells in the 1-mm^2^ area of each plate.

### Animals

Animal experiments and husbandry were approved by the UAA Institutional Animal Care and Use Committee (USDA Assurance #A3710-0) and performed in accordance with US Public Health Service Policy as documented by *The Guide for the Care and Use of Laboratory Animals 8th Edition* [45]. Eight-wk-old male Wistar rats that had been allowed ad libitum access to Mazuri Rodent Diet (PMI Nutrition, St. Louis, MO, USA) were used in the study. Rats were randomly assigned to one of 2 groups and fed for 5 wk with diets based on the Purified AIN76A formulation, modified to Cu content as described by Tallino and coworkers [21]: Cu deficient (<0.3 mg Cu/kg), Cu adequate (125 mg/kg). Briefly, ingredients of diets are 149.1 g casein, 100 g sucrose, 155.9 g maltose dextrin, 465 g corn starch, 40 g cellulose, 35 g salt mix-76-Cu-def, 10 g vit. mix-76A, 3 g Dl-Methionine, 2 g choline bitartrate, 40 g corn oil, and 0 Cu carbonate in Cu-deficient diet and 0.22 g Cu carbonate in Cu-adequate diet. Cu levels were selected consistent with deficient and “normal” Cu diets used by Aigner and coworkers [10]. Thirteen-wk-old rats were killed by carbon dioxide asphyxiation (consistent with 2013 AVMA guidelines) and exsanguinated, and epididymal fat specimens were taken and frozen in liquid nitrogen and then stored at −80°C until use.

### Oil red O staining

Cell monolayers in 6-well plates were washed 2 times with PBS and fixed for 2 min with 4% formaldehyde in PBS. Then, cells were washed twice with water followed by 60% isopropanol for 5 min. The 60% isopropanol was removed completely, and cells were left to dry at room temperature. Stock Oil Red O (0.5%; Sigma, O1391) was diluted with water (3:2), filtered through a 0.45-μm filter, and incubated with dried cells for 2 h at room temperature. Cells were then washed extensively with distilled water, dried, and photographed.

### Mass spectrometry, peptide identification, and quantification

The cell lysates were prepared from the day 0, day 6, and day 9 WT and SSAO^−/−^ 3T3-L1 cells. The samples containing 80 μg protein (pellet after TCA/acetone precipitation) were dissolved in 80 μL 100 mM triethyl ammonium bicarbonate (TEAB). Proteins were reduced by adding 2 μl of 50 mM TCEP at 60°C for 1 h and, after cooling to room temperature, alkylated using 1 μl 200 mM iodoacetamide for 15 min at room temperature. Reduced and alkylated proteins were digested overnight at 37°C by adding 100 μL of 83 ng/μl Trypsin/LysC mixture (V5071, Promega) in 100 mM TEAB. Additionally, the following day, 10 μl of a 20 ng/μl Trypsin/LysC mixture was added to each protein digest for 3 h. The protein digests were then dried and reconstituted in 100 μl 100 mM TEAB prior to labeling the peptides with a unique TMT 10-plex reagent according to the manufacturer’s protocol. The 10 TMT-labeled peptide samples were combined and dried before fractionating over a basic reverse phase (X-Bridge C18, 2.1 × 100-mm, Waters). Fractions were concatenated and combined into 24 fractions and dried.

Approximately 1 μg of each fraction was analyzed by liquid chromatography interfaced with tandem mass spectrometry (LCMSMS) using a Thermo Easy-LC interfaced with a Fusion. Peptides were loaded onto a C18 trap (S-10 μM, 120 Å, 75 μm × 2 cm, YMC, Japan) for 5 min at 5 mL/min in 2% acetonitrile/0.1% formic acid in-line with a 75 μm × 150 mm ProntoSIL-120-5-C18 H column (5 μm, 120 Å (BISCHOFF). Peptides eluting during the 2%–90% acetonitrile in 0.1% formic acid gradient over 100 min at 300 nl/min were directly sprayed into a Fusion mass spectrometer through a 1-μm emitter tip (New Objective, www.newobjective.com) at 2.0 kV. Survey scans (full ms) were acquired from 380–1,600 m/z with a 2-s cycle time, each individually isolated in a 1.0-Da window and fragmented using HCD activation collision energy at 38- and 15-s dynamic exclusion. Precursor and fragment ions were analyzed at resolutions 120,000 and 60,000, respectively, and automatic gain control (AGC) target values at 4e5 with 50-ms maximum injection time (IT) and 1e5 with 118-ms maximum IT, respectively.

Isotopically resolved masses in precursor (MS) and fragmentation (MS/MS) spectra were extracted from raw MS data without deconvolution and with deconvolution using Xtract or MS2 Processor in Proteome Discoverer (PD) software (v1.4, Thermo Scientific). All extracted data were searched using Mascot (2.5.1; www.matrixscience.com) against the 2015RefSeq_complete protein database (*Mus musculus* taxonomy), using the following criteria: precursor mass tolerance of 8 ppm, fragment mass tolerance of 0.03 Da; trypsin as the enzyme, allowing 1 missed cleavage; carbamidomethylation and TMT 10-plex on N-terminus as fixed modifications; methionine oxidation, asparagine, and glutamine deamidation and TMT 6-plex on lysine as variable modifications. Peptide identifications from Mascot searches were filtered at 1% FDR confidence threshold, based on a concatenated decoy database search, using the PD. PD uses TMT reporter ions for quantification only from the peptide identifications with the highest Mascot score from the 3 different extraction methods of the same peptide-matched spectrum (PSM). Protein quantification is based on the normalized median ratio of all spectra of tagged peptides from the same protein [46].

### Analysis of sphingomyelins

A crude lipid extract was obtained using a modified Bligh and Dyer procedure as previously described [47, 48]. As previously described in [49], specifically, sphingomyelin C12:0 (1.3 **μ**g/ml; Avanti Polar Lipids, Alabaster, AL, USA) was included in the extraction solvent as an internal standard. The chloroform layer containing a crude lipid extract was dried in a nitrogen evaporator (Organomation Associates, Berlin, MA, USA) and resuspended in pure methanol. Analyses of sphingomyelins were performed on a triple quadrupole mass spectrometer (API3000, AB Sciex Thornhill, Ontario, Canada) using instrument parameters similar to those described in previous studies [47, 50, 51]. Samples were injected using an Agilent 1100 high-pressure liquid chromatography (HPLC) (Agilent Technologies, Santa Clara, CA, USA) equipped with a reverse phase C18 column (Phenomenex, Torrance, CA, USA). Sphingomyelin species were separated by gradient elution at the flow rate of 0.7 mL/min. Mobile phases consisted of (A) 60% methanol, 39% H_2_O, 1% formic acid with 5 mM ammonium formate and (B) 99% methanol, 1% formic acid, and 5 mM ammonium formate. Gradient conditions were as follows: 60% B for 0.01 min, a gradual increase to 100% B over the next 0.49 min, and hold at 100% B for 3 min; decline from 100% to 0% B during the next 0.01 min, hold at 0% B for 0.99 min, increase from 0% to 60% B for 0.5 min, and hold at 60% B for the final 0.5 min. The eluted sample was injected into the ion source, where the detection of each sphingomyelin species was conducted by multiple reaction monitoring in positive mode. The ion spray voltage (V) was 5,500 at a temperature of 80°C with a nebulizer gas of 9 psi and curtain gas of 8 psi, and the collision gas set at 10 psi. The declustering potential was 60 V, the focusing potential 300 V, the entrance potential 10 V, the collision energy 30 V, and the collision cell exit potential 10 V. MS/MS scanned from 300 to 1,000 atomic mass units (amu) per second with steps of 0.1 amu. Eight-point calibration curves (0.1 to 1,000 ng/ml) were constructed by plotting the area under the curve for sphingomyelin C16:0, C18:0, C20:0, C22:0, and C24:0 (Avanti polar lipids, Alabaster, AL, USA) prepared in pure methanol normalized to the C12:0 internal standard. Concentrations of sphingomyelins in each sample were determined by fitting the identified sphingomyelin and dihydrosphingomyelin species to these standard curves based on acyl-chain length. Intraday coefficients of variation (CVs) for each sphingomyelin species were less than 10% [49]. Instrument control and quantification were performed using Analyst 1.4.2 and MultiQuant software (AB Sciex Thornhill, Ontario, Canada).

### Bioinformatic analysis

Multivariate statistics and associated graphics were generated using the R programming tool for plotting data version 3.4.1. Heat maps were drawn using the heatmap.2 function found in the gplots package. The associations between the altered proteins and major metabolic pathways were identified using the Ingenuity Pathways Analysis software (Ingenuity Systems, www.ingenuity.com). Differentially expressed proteins (a fold change of 1.5 or higher) and their abundance values were used for the analysis.

### Statistical analysis

Statistical analysis was performed using Prism software version 6.0b (GraphPad Software). Two-tailed Student’s *t* tests were used when appropriate. All data in the figures are shown as the mean ± SEM. A *p*-value of less than 0.05 was considered significant.

## Supporting information

S1 Fig Protein levels of ATP7A and ATP7B in differentiated 3T3-L1 adipocytes. Representative western blot showing protein levels of ATP7B and ATP7A in differentiated 3T3-L1 adipocytes. Na+/K+ ATPase (Na/K) is a loading control (*n* = 2). Related to Fig 1.
(TIFF)

S2 Fig Levels of mRNA of SSAO and ATP7A in Cu-deficient and ATP7A^+/−^ adipocytes. (A) Relative mRNA levels of SSAO and ATP7A in adipocytes in basal medium and in TTM (*n* = 3), normalized to basal condition. (B) The mRNA levels of SSAO and ATP7A in the WT and ATP7A^+/−^ adipocytes (*n* = 3), normalized to basal condition. Underlying data can be found in S1 Data; Student’s *t* test, ****p < 0.0001, ***p < 0.001, **p < 0.01, *p < 0.05, ns *p* > 0.05. The data are presented as mean ± SEM; related to Fig 3. Cu, copper; ns, not significant; SSAO, semicarbazide-sensitive amine oxidase; TTM, tetrathiomolybdate; WT, wild-type.
(TIFF)

S3 Fig Cellular localization of SSAO in differentiated WT and ATP7A^+/−^ adipocytes. Immunocytochemistry of differentiated WT and ATP7A^+/−^ adipocytes with anti-SSAO antibodies shows similar intracellular distribution and targeting of SSAO to the plasma membrane; low magnification images (upper panels) and high magnification images (lower panels) are shown. Related to Fig 3. SSAO, semicarbazide-sensitive amine oxidase; WT, wild-type.
(TIFF)

S4 Fig Soluble SSAO does not significantly affect the WT adipocytes when present in the medium during adipogenesis. (A) Cell size distribution and (B) triglyceride levels in adipocytes grown in the basal medium (*n* = 113, *n* = 3) or in the same medium supplemented with 1 μg/ml sSSAO (*n* = 103, *n* = 3) from day 0 to day 8. Underlying data can be found in S1 Data; Student’s *t* test, ****p < 0.0001, ***p < 0.001, **p < 0.01, *p < 0.05, ns *p* > 0.05. The data are presented as mean ± SEM and median ± IQR for cell distribution; related to Fig 4. ns, not significant; SSAO, semicarbazide-sensitive amine oxidase; sSSAO, recombinant soluble SSAO; WT, wild-type.
(TIFF)

S5 Fig Soluble SSAO does not significantly affect SSAO^−/−^ adipocytes, if added post differentiation. (A) Cell size distribution and (B) triglyceride levels for differentiated SSAO^−/−^ adipocytes in the basal medium (*n* = 130, *n* = 3) or after treatment with 1 μg/ml sSSAO (*n* = 142, *n* = 3) from day 8 to day 16. Underlying data can be found in S1 Data; Student’s *t* test, ****p < 0.0001, ***p < 0.001, ** < 0.01, *p < 0.05, ns *p* > 0.05. The data are presented as mean ± SEM and median ± IQR for cell distribution; related to Fig 4. ns, not significant; SSAO, semicarbazide-sensitive amine oxidase; sSSAO, recombinant soluble SSAO; WT, wild-type.
(TIFF)

S1 Table Proteins deregulated in SSAO^−/−^ cells and rescued by incubation with a recombinant soluble SSAO. SSAO, semicarbazide-sensitive amine oxidase.
(XLSX)

S2 Table Ratios of proteins associated with fatty acid uptake in cells with and without SSAO. Protein abundances were determined by mass spectrometry, as described in the Methods section; the pathways were identified using Ingenuity pathways. The proteins associated with fatty acid uptake and altered abundance were identified for each time point, and ratios were generated. SSAO, semicarbazide-sensitive amine oxidase
(TIFF)

S3 Table Primers used in these studies for mRNA analysis by real-time PCR. (TIFF)

S1 Data Original numerical data of figures. (XLSX)

S2 Data Raw proteomics dataset. (XLSX)

## Abbreviations

ABCD1: ATP-binding cassette subfamily D member 1
ACSL: acyl-coenzyme A synthase
ACSL1: acyl-coenzyme A synthetase long-chain family member 1
ACSL4: acyl-coenzyme A synthetase long-chain family member 4
ACSL5: acyl-coenzyme A synthetase long-chain family member 5
ACSL6: acyl-coenzyme A synthetase long-chain family member 6
AGC: automatic gain control; amu, atomic mass unit; Ano10, anoctamin 10
AOC3: amino-oxidase copper-containing 3
Atox1: antioxidant 1 Cu chaperone; BCS, bathocuproine disulfonate
Cas9: CRISPR-associated 9
CCS: copper chaperone for superoxide dismutase
CERS6: ceramide synthase 6
CHPT1: choline phosphotransferase 1
CRISPR: clustered regularly interspaced short palindromic repeat
CTR1: copper transporter 1
CTR2: copper transporter 2
Cu: copper
CV: coefficient of variation
DMEM: Dulbecco’s Modified Eagle Medium
DMI: differentiation medium I
DMII: differentiation medium II
ECAO: *E*. *coli* copper amine oxidase
ENO1: enolase 1
FATP: fatty acid transport protein
FBS: fetal bovine serum
FDR: false discovery rate
FVT1: follicular variant translocation protein 1
GLAP: glyceraldehyde 3-phosphate
HPLC: high-pressure liquid chromatography
IBMX: 3-Isobutyl-1-methylxanthine
IT: injection time
KO: knockout
LCMSMS: liquid chromatography interfaced with tandem mass spectrometry
MAO-B: monooxygenase B
NAFLD: nonalcoholic fatty-liver disease
ns: not significant
OA: oleic acid
OSTC: oligosaccharyltransferase complex
PAM: protospacer-adjacent motif
PD: Proteome Discoverer
PFKL: phosphofructokinase, liver type
PGAM1: phosphoglycerate mutase 1
PGK1: phosphoglycerate kinase 1
PI3K: phosphoinositide 3-kinase
PKM: pyruvate kinase
PPARγ: peroxisome proliferator–activated receptor gamma
PSM: peptide-matched spectrum
RT-PCR: real-time PCR
sgRNA: single guide RNA
SiN: silicon nitride
SMPD2: sphingomyelin phosphodiesterase 2
SMPD3: sphingomyelin phosphodiesterase 3
SOD1: superoxide dismutase 1
SOD3: superoxide dismutase 3
SPTLC2: serine palmitoyltransferase long chain base subunit 2
SSAO: semicarbazide-sensitive amine oxidase
sSSAO: recombinant soluble SSAO
Syn6: syntaxin 6
TEAB: triethyl ammonium bicarbonate
TGN: *trans*-Golgi network
THBS1: thrombospondin 1
TMT: tandem mass tag
TTM: tetrathiomolybdate
UAA: University of Alaska Anchorage
VAP-1: vascular adhesion protein 1
VLDL: very low-density lipoprotein
WT: wild type
XFM: X-ray fluorescence microscopy
